# *Sargassum Fusiforme* Polysaccharide SFP-F2 Activates the NF-κB Signaling Pathway via CD14/IKK and P38 Axes in RAW264.7 Cells

**DOI:** 10.3390/md16080264

**Published:** 2018-08-01

**Authors:** Liujun Chen, Peichao Chen, Jian Liu, Chenxi Hu, Shanshan Yang, Dan He, Ping Yu, Mingjiang Wu, Xu Zhang

**Affiliations:** 1 College of Life and Environmental Science, Wenzhou University, Wenzhou 325035, China; lchen3@unbc.ca (L.C.); chenpeichao@wzu.edu.cn (P.C.); 15451382280@stu.wzu.edu.cn (J.L.); 15451382279@stu.wzu.edu.cn (C.H.); 14112822281@stu.wzu.edu.cn (S.Y.); 13112822263@stu.wzu.edu.cn (D.H.); yupingwzu@gmail.com (P.Y.); 2 Natural Resources and Environmental Studies Program, University of Northern British Columbia, Prince George, BC V2N 4Z9, Canada

**Keywords:** *Sargassum fusifrome*, polysaccharides, NF-κB signaling pathway, CD14, P38, RAW264.7 cell

## Abstract

*Sargassum fusifrome* is considered a “longevity vegetable” in Asia. *Sargassum fusifrome* polysaccharides exhibit numerous biological activities, specially, the modulation of immune response via the NF-κB signaling pathway. However, the precise mechanisms by which these polysaccharides modulate the immune response through the NF-κB signaling pathway have not been elucidated. In this study, we purified and characterized a novel fraction of *Sargassum fusifrome* polysaccharide and named it SFP-F2. SFP-F2 significantly upregulated the production of the cytokines TNF-α, IL-1β and IL-6 in RAW264.7 cells. It also activated the NF-κB signaling pathway. Data obtained from experiments carried out with specific inhibitors (PDTC, BAY 11-7082, IKK16 and SB203580) suggested that SFP-F2 activated the NF-κB signaling pathway via CD14/IKK and P38 axes. SFP-F2 could therefore potentially exert an immune-enhancement effect through inducing the CD14/IKK/NF-κB and P38/NF-κB signaling pathways.

## 1. Introduction

*Sargassum fusiforme* (*S. fusiforme*) is a brown alga commonly found along the rocky coastlines of Korea, Japan and China (especially in Fujian and Zhejiang provinces). For thousands of years, *S. fusiforme* has been used as a traditional Chinese medicinal herb to treat thyroid diseases. Moreover, *S. fusiforme* also features in Asian diets as a health promoting food. Growing evidence indicates that *S. fusiforme* contains abundant nutritional and pharmaceutical ingredients [1,2]. However, the underlying mechanism associated with the immuno-stimulating activity of *S. fusiforme* polysaccharides has remained largely undetermined.

Polysaccharides isolated from animals, plants, fungi, and bacteria can have immune enhancement effects. Accumulated evidence shows that polysaccharides extracted from *S. fusiforme* exhibit numerous biological activities, including anti-tumor, anti-oxidation, anticoagulant, immuno-stimulating and anti-aging activities [3,4,5]. Among these, the immuno-stimulating activity of the polysaccharides has been widely studied, and it has been shown to increase the weight of the thymus and spleen [6], alter gene expression in peritoneal macrophages [7], enhance the growth of lymphocytes, and promote the productions of IgG and inflammatory cytokines [8,9]. *S. fusiforme* polysaccharide exerts its immuno-stimulating activity through the TLRs (toll-like receptors)/NF-κB (nuclear factor kappa-light-chain-enhancer of activated B cells) signaling pathway [9]. In this study, a novel polysaccharide fraction was isolated from *S. fusiforme*. The physicochemical properties of this polysaccharide, including the molecular weight, monosaccharide compositions, glycosidic bonds, and sulfated and uronic acid contents were characterized. Furthermore, the immuno-stimulating activity of the polysaccharide was also investigated using a mouse macrophage cell line. The aim of this study was to obtain more insight into the molecular mechanism that *S. fusiforme* polysaccharide manifests its immuno-stimulating activity.

## 2. Results

### 2.1. Physicochemical Characterization of SFP-F2

**Figure 1:**
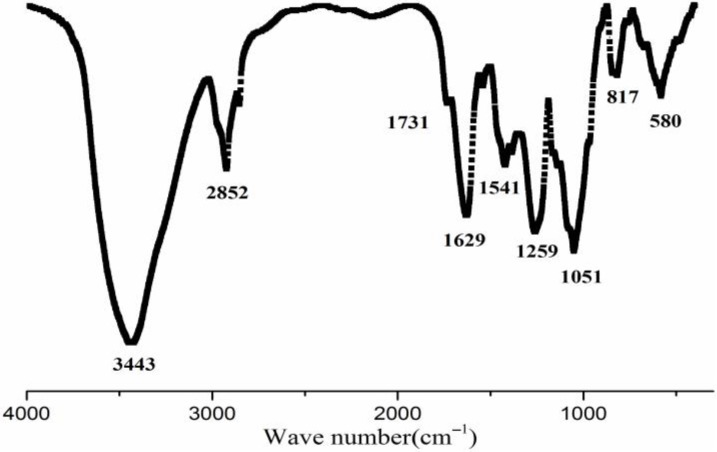
Fourier-transform infrared spectroscopy (FT-IR) spectrum of SFP-F2

A crude preparation of *S. fusiforme* polysaccharide was obtained by hot water extraction and ethanol precipitation followed by decoloration via DEAE-cellulose chromatography. This preparation was designated as SFP, and it was further purified by DEAE Sepharose CL-6B chromatography, and then by Sephacryl^TM^ S-400 HR chromatography to yield the final product, designated as SFP-F2. The chemical composition of SFP-F2 is shown in Table 1. The content of SFP-F2 contained more than 60% sugar and about 27.7% sulfate. Five monosaccharides were found in SFP-F2 (Table 1). Fucose made up the largest proportion (>80%) followed by galactose (13.3%). The rest of the monosaccharides accounted for less than 7% of the sugar. SFP-F2 had an average molecular weight of 24 kDa as determined by HPLC. Fourier-transform infrared spectroscopy (FT-IR) spectrum of SFP-F2 spectral analysis of SFP-F2 revealed a wide band at 3600~3200 cm^−1^, representing hydroxyl stretching vibration absorption (Figure 1), and a carbonyl band at 1641 cm^−1^ was a characteristic feature of polysaccharides. Intense absorption at 2800~2912 cm^−1^ might indicate a C-H bend and stretching vibration of a pyranoid ring or even the C-6 of fucose and galactose. The band at 1731 cm^−1^ was assigned to the C=O stretching vibration of *O*-acetyl groups [10]. The absorption at 1541 cm^−1^ might indicate the presence of scissoring vibrations of CH_2_ (galactose, xylose) and asymmetric bending vibrations of CH_3_ (fucose, *O*-acetyls) [11]. The band at 1259 cm^−1^ was assigned mainly to the asymmetric O=S=O stretching vibration of sulfate esters. The absorption peak at 1051 cm^−1^ could possibly be contributed by the C3-O-C6 bridge of the anhydrogalactose residue [12]. The absorption at 817 cm^−1^ suggested the presence of sulfate groups at the equatorial C-2 and C-3 positions [13]. The band at 580 cm^−1^ suggested the obvious asymmetric deformation absorption of O-S-O, which is characteristic of sulfate content and the substitution position.

### 2.2. Effect of SFP-F2 on Cytokine Production in RAW264.7 Cells

**Figure 2:**
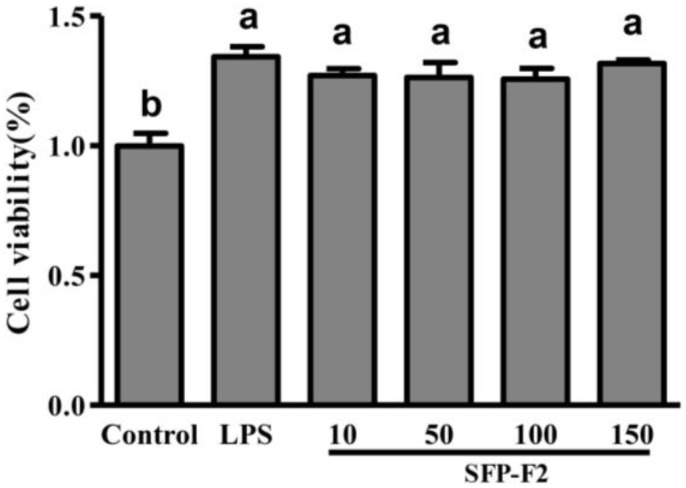
Effect of SFP-F2 on the viability of RAW 264.7 cells. RAW 264.7 cells were seeded in 96-well plate and cultured until 70–80% confluency. The cells were then treated with 1 μg/mL lipopolysaccharides (LPS) or with different concentrations of SFP-F2 in DMEM medium for 12 h. The cells were then subjected to MTT assay to assess their viability. Data are presented as the means ± SDs from three independent experiments. Means with different alphabet (a, b) indicate a significant difference at *p* < 0.05 on the cell viability of polysaccharides between groups with the Tukey-Kramer test.

SFP-F2 caused no loss of cell viability to RAW264.7 cells at the range of concentrations (10–150 μg/mL) tested (Figure 2). In fact, RAW264.7 cells treated with SFP-F2 displayed a significant increase in viability compared to the control (non-treated) cells. Likewise, RAW264.7 cells treated with 1 μg/mL lipopolysaccharides (LPS) also exhibited a significant increase in cell viability over the control cells. Since arsenic is one of the common toxic heavy metals in seaweeds. The result clearly showed that SFP-F2 enhanced the viability of RAW264.7 cells. The safety of *S. fusiforme* polysaccharides was indirectly confirmed by in vivo experiments done on ICR mice by intragastric administration with crude polysaccharides [4,5].

**Figure 3:**
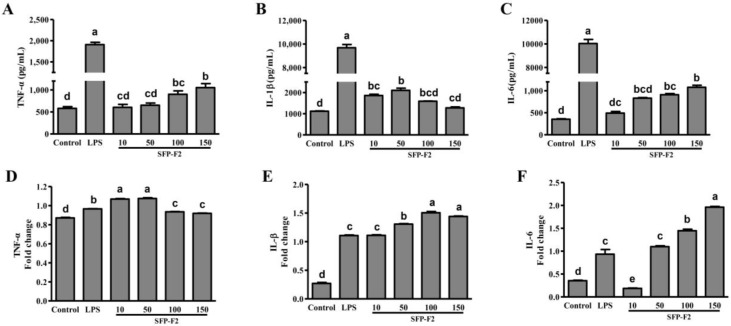
SFP-F2 modulates cytokine release by RAW 264.7 Cells. RAW264.7 cells (in DMEM) were seeded in a 96-well plate (100 μL/well) and cultured until 70–80% confluency. This was followed by the addition of 100 μL DMEM only (blank control) or DMEM containing 1 μg/mL LPS (positive control) or different concentrations of SFP-F2 and an additional 12 h-incubation. The levels of TNF-α (**A**), IL-1β (**B**), IL-6 (**C**) were assayed by ELISA whereas the mRNA expression levels of TNF-α (**D**), IL-1β (**E**), IL-6 (**F**) were assayed by reverse transcription-polymerase chain reaction RT-PCR. Data are presented as the means ± SDs from three independent experiments. Means with different alphabet (a-e) indicate a significant difference at *p* < 0.05 between groups with the Tukey-Kramer test.

The effect of SFP-F2 on cytokines released by RAW264.7 cells following treatment with the polysaccharide was determined by ELISA. TNF-α levels were significantly increased by treatment with SFP-F2 at 100 μg/mL and 150 μg/mL (Figure 3A). Similarly, the levels of IL-1β and IL-6 were also increased by treatment with SFP-F2 (Figure 3B,C), with IL-1β level peaked at 50 μg/mL SFP-F2. LPS (used as positive control) had a much stronger stimulating effect on the translational levels of TNF-α, IL-1β and IL-6 in RAW264.7 cells compared to SFP-F2. The effect of SFP-F2 on the transcript levels of TNF-α, IL-1β and IL-6 in RAW264.7 cells was also investigated by reverse transcription PCR assay, which revealed significant increase in the transcript levels of the three cytokines in the cells treated with SFP-F2 compared to the control cells. Taken together, the results showed that treatment of RAW264.7 cells with SFP-F2 led to increased secretions of immune response-related cytokines.

### 2.3. SFP-F2 Stimulates the NF-κB Signaling Pathway

Treatment of RAW264.7 cells with SFP-F2 resulted in an immune-related response manifested at both the transcriptional and translational levels. The effect of SFP-F2 on the immune-related NF-κB signaling pathway was assessed by detecting the phosphorylation levels of the IκBα and P65 in RAW264.7 cells.

**Figure 4:**
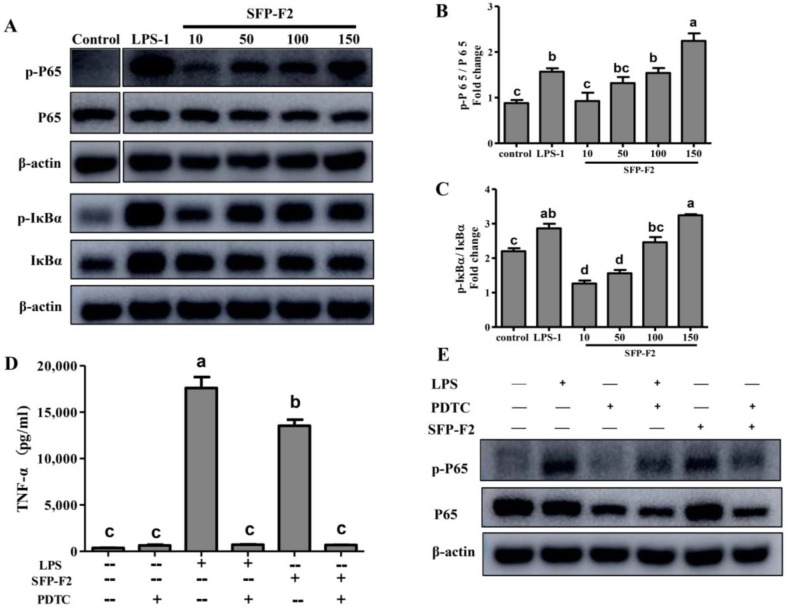
Modulation of the NF-κB signaling pathway in RAW 264.7 cells by treatment with SFP-F2. RAW264.7 cells were seeded in 10-cm dishes and cultured until 70–80% confluency. The cells were then treated with medium only or medium containing with 1μg/mL LPS or different concentrations of SFP-F2 for 12 h. The levels of IκBα, p-IκBα and NF-κB (P65), p-P65 in the cells were assayed by western blot. (**A**) Representative blots showing the effect of SFP-F2 on each of the proteins examined. (**B**,**C**) Comparison of the levels of the phosphorylated protein relative to their non-phosphorylated counterparts in grey scale. (**D**) Effect of SFP-F2 on the secretion of TNF-α in the presence of PDTC. The cells were incubated with 100 μM PDTC for 2 h followed by incubation with 150 μg/mL SFP-F2 for another 3 h. The level of TNF-α in the culture supernatant was assayed by ELISA. (**E**) Western blots showing the changes in the level of phosphorylated P65 to non-phosphorylated P65 in cells. Data are presented as the means ± SDs from three independent experiments. Means with different alphabet (a–d) indicate a significant difference at *p* < 0.05 between groups with the Tukey-Kramer test.

SFP-F2 upregulated the phosphorylation of the P65 (p-P65) and IκBα (p-IκBα) in a dose-dependent manner (Figure 4A). RAW264.7 cells treated with 150 μg/mL SFP-F2 exhibited a significant increase in p-P65/P65 and p-IκBα/IκBα ratios (Figure 4B,C). Furthermore, as shown in Figure 4C, PDTC significantly suppressed the expression level of TNF-α even in the presence of LPS or SFP-F2. Both P65 and p-P65 were significantly down-regulated by PDTC treatment (Figure 4D). The data suggested that the immuno-stimulating effect of SFP-F2 was suppressed by PDTC. Collectively, the results indicated that SFP-F2 might activate the NF-κB signaling pathway in RAW264.7 cells via induction of P65 phosphorylation.

### 2.4. CD14/IKK Signaling is Involved in the Immunomodulatory Effects of SFP-F2

**Figure 5:**
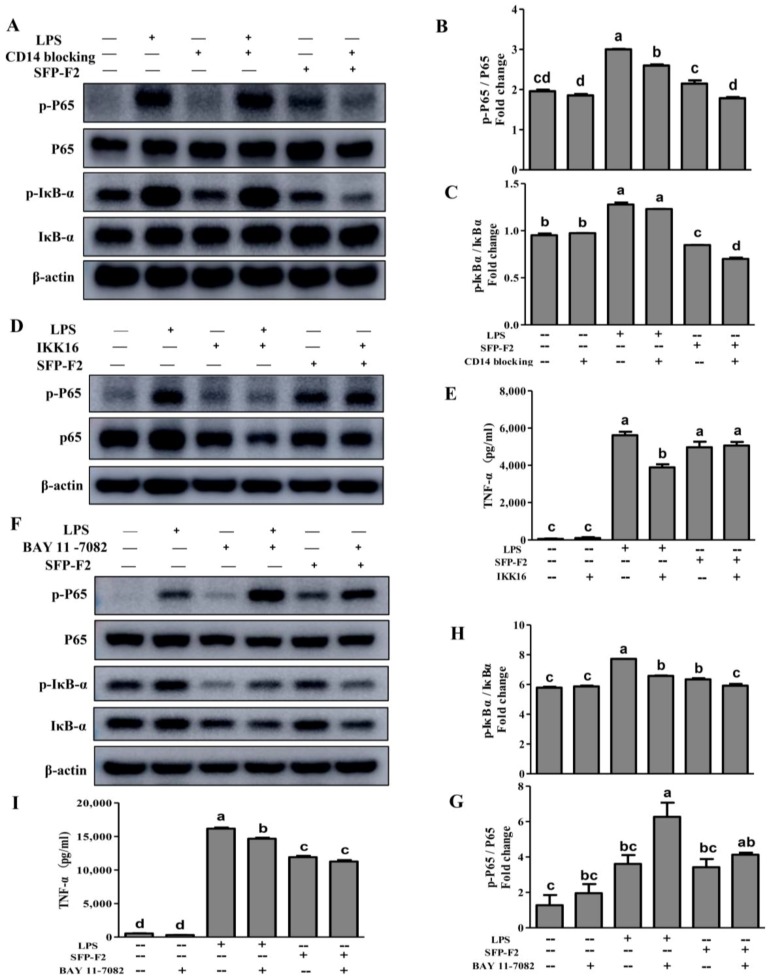
Modulation of CD14/IKK signal transduction by SFP-F2. RAW 264.7 cells were seeded in 10-cm dishes, cultured until 70–80% confluency, and then incubated with 1 μg/mL CD14 antibody (**A**), 10 μM IKK-16 (**D**) and or 10 μM BAY 11-7082 (**F**) for 2 h followed by further incubation without or with 1 μg/mL LPS or 150 μg/mL SFP-F2 in the presence of different inhibitors for another 3 h. Control cells were treated with complete medium containing 0.02% DMSO only. Representative blots are shown. Comparison of the levels of phosphorylated protein relative to the levels of their non-phosphorylated counterparts in grey scale: p-P65/P65 (**B**,**G**) and p-IκBα/IκBα (**C**,**H**). The levels of TNF-α released by IKK16-treated (**E**) or BAY 11-7082-treated (**I**) cells in the absence or presence of SFP-F2. Data are presented as the means ± SDs from three independent experiments. Means with different alphabet (a-d) indicate a significant difference at *p* < 0.05 between groups with the Tukey-Kramer test.

CD14, a 55-kDa GPI-anchored protein, is expressed on the surface of monocytes, neutrophils and macrophages [14]. CD14 is highly sensitive to LPS, which serves as a co-receptor for the complex formed by LPS and LPS-binding protein (LBP) [15], as it can facilitate the synthesis of TNF-α, IL-6 and IL-8 [16] via activation of the NF-κB signaling pathway [17,18]. Blocking CD14 with a CD14-blocking antibody in the presence of SFP-F2 led to significant decrease in the levels of p-IκBα and p-P65 (Figure 5A–C), suggesting that CD14, a receptor for carbohydrate ligands, was obviously involved in the immuno-stimulating effect of SFP-F2.

To further explore the underlying canonical NF-κB signaling in response to the influence of SFP-F2, a series of specific inhibitors were used. IKK16, an inhibitor of the IKK complex, showed no effect on the phosphorylation of P65 or the secretion of TNF-α (Figure 5D,E) in the presence of SFP-F2. Subsequent blocking of the phosphorylation of IκBα (a downstream protein of the IKK complex) with BAY 11-7082 in the presence of SFP-F2 did not change the level of P65 or p-P65 (Figure 5F,G). Instead, it caused a slight increase in the level of p-P65 (Figure 5F), while abolishing both the expression of IκBα and the phosphorylation of IκBα (Figure 5F,H). In addition, BAY 11-7082 showed a slight suppression effect (*p* > 0.05) on the SFP-F2-induced secretion of TNF-α (Figure 5I). Thus, in addition to the CD14/IKK/IκBα axis, SFP-F2 might activate another signaling pathway to regulate the expression of TNF-α when the canonical NF-κB signaling is blocked.

### 2.5. SFP-F2 Stimulates P38/NF-κB Signaling Transduction in RAW264.7 Cell

**Figure 6:**
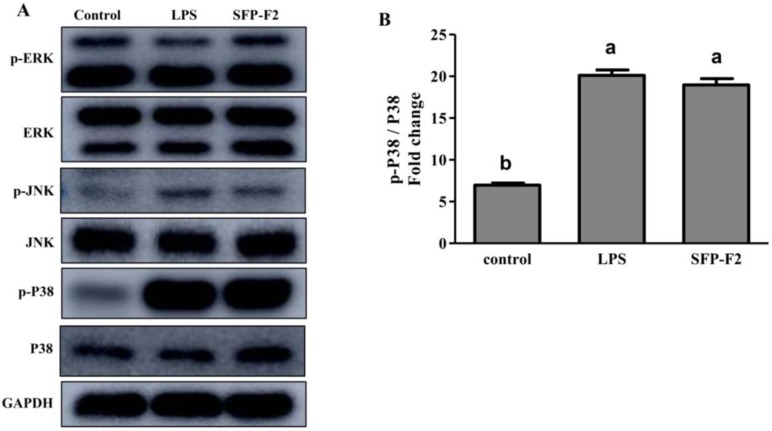
Selective activation of P38 in the MAPK signaling pathway induced by SFP-F2. RAW 264.7 cells were seeded in 10-cm dishes and cultured until 70–80% confluency, and then treated without (control) or with 1 μg/mL LPS or 150 μg/mL SFP-F2 for 12 h. (**A**) Blot showing the levels of phosphorylated and non-phosphorylated proteins in response to LPS and SFP-F2 treatments. (**B**) Comparison of the levels of phosphorylated P38 relative to the levels of non-phosphorylated P38 in grey scale (**B**). Data are presented as the means ± SDs from three independent experiments. Means with different alphabet (a, b) indicate a significant difference at *p* < 0.05 on p-P38/P38 ratio between groups with the Tukey test.

**Figure 7:**
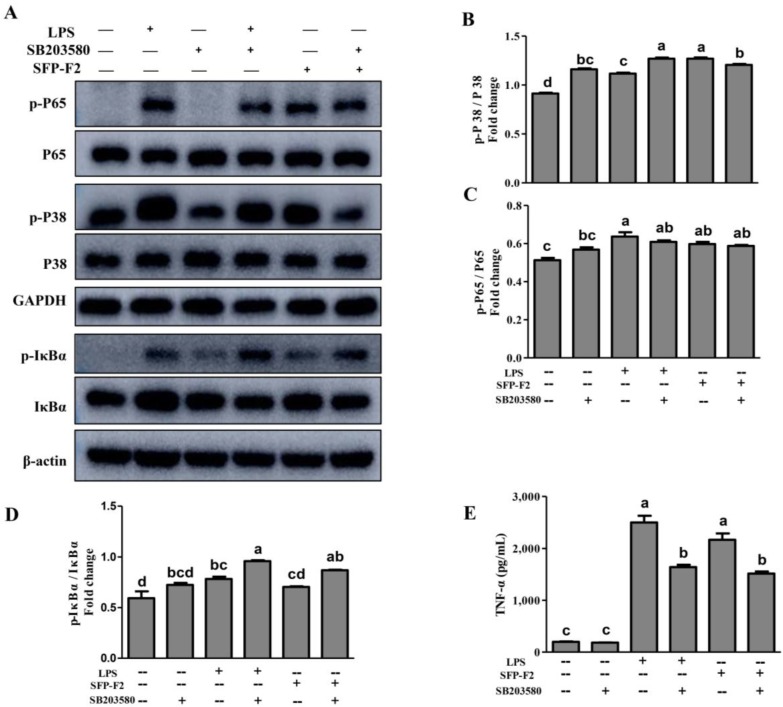
SFP-F2 Stimulates P38/NF-κB signal Transduction in RAW 264.7 Cells. RAW 264.7 cells were seeded in 10-cm dishes and cultured until 70–80% confluency, and then treated with 2 μM SB203580 for 2 h followed by treatment without or with 1 μg/mL LPS or 150 μg/mL SFP-F2 for another 3 h. (**A**) Western blot analysis of the different key proteins involved in the P38/NF-κB signaling pathways. Representative blots are shown. (**B**–**D**) The plots compare the levels of phosphorylated proteins relative to the level of their non-phosphorylated counterparts in grey scale. (**E**) ELISA assay of the TNF-α released by cells that had been treated with 2 μM SB203580 in the absence or presence of SFP-F2. Data are presented as the means ± SDs from three independent experiments. Means with different alphabet (a–d) indicate a significant difference at *p* < 0.05 between groups with the Tukey-Kramer test.

Macrophages are the primary effector cells that interact with the CD14 receptor and activate the NF-κB/mitogen-activated kinase (MAPK) signaling pathway in the innate immune response [19]. The MAPK cascade pathways response to extracellular signals by activating various transcription factors and gene expression [20,21]. JNK, ERK, and P38 are the main members of the MAPK family [22]. RAW264.7 cells treated with SFP-F2 or LPS showed remarkable up-regulation of P38 phosphorylation, with a slight increase in expression of JNK (Figure 6A,B), suggesting that P38 was more sensitive to stimulation by SFP-F2. However, in the presence of SB203580, the upregulation of P38 phosphorylation induced by SFP-F2 was significantly suppressed (Figure 7A,B). Furthermore, the level of secreted TNF-α was dramatically decreased (Figure 7E) corresponding to a slightly reduced phosphorylation level of P65 (Figure 7C). Despite being treated with SFP-F2 and SB203580, an increase in IκBα phosphorylation was still observed (Figure 7A). Intriguingly, the results demonstrated that IκBα was not intensively regulated by P38 in the presence of SFP-F2, which further confirmed that IκBα deficiency did not affect the activation of the NF-κB signaling pathway by SFP-F2 (Figure 5). More importantly, P38 seemed to play a role of an upstream regulatory protein of p65 in the immune-stimulating effect of SFP-F2.

### 2.6. SFP-F2 Promotes an Immune Response in RAW264.7 Cells Via a Combined P38/NF-κB Signaling Transduction

**Figure 8:**
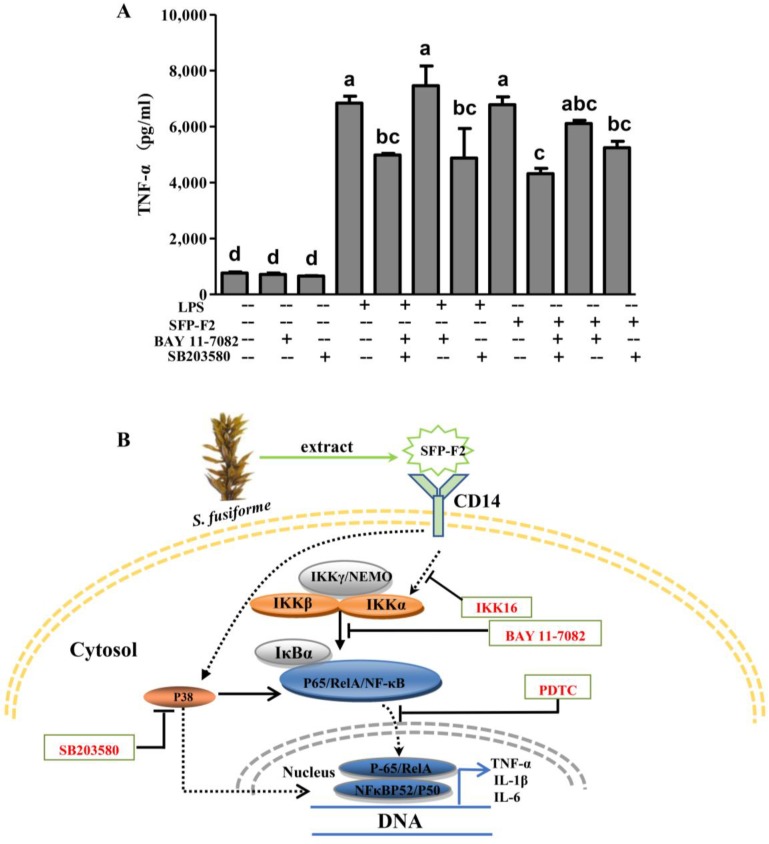
Effect of SFP-F2 on TNF-α release by RAW264.7 cells in the presence of inhibitors against the P38/NF-κB Signaling pathways. (**A**) ELISA assay of TNF-α released by RAW264.7 cells treated with SFP-F2 in the absence and presence of the inhibitors. RAW264.7 cells were seeded in 10-cm dishes and cultured until 70–80% confluency, and then treated with 2 μM SB203580 plus 10 μM BAY 11-7082 for 2 h followed by treatment without or with 1μg/mL LPS or 150 μg/mL SFP-F2 for another 3 h. (**A**) TNF-α levels secreted by the cells from different treatments as determined by ELISA. Data are presented as the means ± SDs from three independent experiments. Means with different alphabet (a-d) indicate a significant difference at *p* < 0.05 between groups with the Tukey-Kramer test. (**B**) The proposed mechanism for the immuno-stimulating activity of SFP-F2 in RAW264.7 cells. Solid arrow indicates direct stimulatory modification; dotted arrow indicates tentative stimulatory modification (in the cytosol) and translocation (in the nucleus); blocked arrow indicates direct inhibitory modification.

To determine whether the effect of SFP-F2 could be down-regulated by blocking both P38 MAPK and NF-κB signaling pathways, RAW264.7 cells were treated with both BAY 11-7082 and SB203580 plus SFP-F2, and the levels of TNF-α were measured. The result showed that co-treatment with BAY 11-7082 and SB203580 markedly reduced the secretion of TNF-α promoted by SFP-F2 or LPS (Figure 8A). These observations indicated that activation of the NF-κB signaling pathway by SFP-F2 might depend more on the P38 pathway than the IKK complexes/IκBα pathway, with the consequent upregulation of cytokines such as TNF-α, consistent with the data in Figure 5E,I.

## 3. Discussion

Although *S. fusiforme* has been consumed as a longevity vegetable in Asia, much still remains to be determined in terms of the underlying mechanisms. It is assumed that *S. fusiforme* polysaccharides exhibit different biological activities and are intensively associated with their structural features. Growing evidences suggest that the degree of sulfatation in polysaccharides is positively related to their immuno-stimulating activities [3,23]. Since SFP-F2 contained a higher percentage of sulfates (27.7%) than the crude polysaccharide extract (22.3%), we speculated that it might possess potential immuno-stimulating activity. The upregulation of TNF-α, IL-1β and IL-6 productions in RAW264.7 cells induced by SFP-F2 (Figure 3) strongly supported this speculation, especially since these cytokines are commonly secreted by the immune cells in response to exogenous stimulation.

Macrophages play an indispensable role in the immune response in the living organism. In addition to directly eliminating pathogens by phagocytosis, these cells also secrete chemokines and immune factors [24,25], which are capable of initiating the innate immune responses, especially in adaptive immunity [26], eventually leading to the clearance of pathogens. Cytokines as TNF-α, IL-6 and IL-1β are essential for immuno-regulation. For example, IL-6 is involved in the regulation of B cell and T cell proliferation and differentiation [27], whereas IL-1β can promote bone resorption, fever, induction of prostaglandin synthesis, and augment the responses of T-cell to an antigen. TNF-α is a pro-inflammatory cytokine that induces bone resorption and up-regulates prostaglandin E2 (PGE2) and Matrix metalloproteinase (MMP) secretion [28]. The immuno-stimulating effect of SFP-F2 was evident from the induced expression of TNF-α, IL-1β and IL-6 in SFP-F2-treated RAW264.7 cells (Figure 3). Although the increase at the transcriptional level did not parallel with the increase at the translational level, we presumed that intracellular mRNA was degraded once it was synthesized, while the translated product continued to accumulate in the medium.

Further, these cytokines are regulated by NF-κB signaling acting as part of the immune responses against the pathogens. NF-κB, a dimer of transcription factors, which are primarily consisting of P65 (RelA) or P50, and they play critical roles in the triggering and coordination of both innate and adaptive immune responses [29]. P65 is the transcriptional activation component of the most common form of the NF-κB & P50 heterodimer. In quiescent cell, NF-κB and P50 form an inactive heterodimer, anchored by IκBα and IκBβ, which function to retain NF-κB in the cytosol [30]. Phosphorylation of IκBα at Ser^32^ and Ser^36^, and the phosphorylation of IκBβ at Ser^19^ and Ser^23^, which are targeted by the IKKβ subunit of the IKK complex in the course of the canonical NF-κB signaling [31], leading to the release releases of active NF-κB, which then translocates to the nucleus, binds to specific DNA enhancer sequences (κB binding sites), and activates pro-inflammatory target gene transcription [32]. Chen et al. reported that *S. fusiforme* polysaccharides can stimulate the secretion of cytokines via the TLR2/4 and CD14 receptors and subsequent activation of P65, measured as an increase in nuclear P65 following polysaccharide treatment [9]. However, less information concerning the detail analysis of the relationship between the polysaccharides and the key proteins involved in the NF-κB/MAPK signaling pathway in the presence of *S. fusiforme* polysaccharides. Our data appeared to indicate that the immuno-stimulating activity of SFP-F2 also involves the activation of IKKs, IκBα and P38 MAPK. Blocking CD14 with antibody inhibited the phosphorylation of IκBα and P65, confirming that CD14, which is a potential membrane co-receptor for carbohydrate ligands, participated in the SFP-F2-stimulated immune response via the NF-κB pathway. IKK16 and BAY 11-7082 are specific inhibitors used to block the activity of the IKK complex [33] and the phosphorylation of IκBα, respectively. The IKK complex (IKKα and IKKβ) known as the IκB kinase, is a critical regulatory protein in the phosphorylation of IκBs linked to the canonical NF-κB activation mechanism. Both inhibitors of IKK complex and IκBα failed to suppress the SFP-F2-stimulated phosphorylation of P65 and secretion of TNF-α secretion. Serine 536 in the C-terminal transactivation domain of P65 has been shown to boost the TNF-α-induced DNA binding activity of P65 and the recruitment of p300 to the P65/P50 complex, while blocking the phosphorylation of P65 at Ser 536 can abolish the transactivation activity of P65 [34,35]. Through the use of specific inhibitors, we were able to demonstrate that SFP-F2 exerts its immuno-stimulating activity not only through the IKK complex and IκBα in the canonical NF-κB pathway, but also through other pathways via the activation of P65 phosphorylation.

MAPK has been considered as a superfamily involved in the initiation of the NF-κB signaling pathway [21]. Recent reports suggest that ERK is usually associated with growth-promoting mitogenic stimuli, while JNK and P38 are mainly associated with the immune activities [36]. The phosphorylation of mammalian P38 is dependent on CD14, which responds to LPS stimulation [37]. Evidence further suggests that P38 MAPK and NF-κB are sensitive to similar stimulations. In addition, P38 MAPK can play an indispensable role in NF-κB-dependent gene expression [21,38] via the phosphorylation of IκBα, nuclear translocation of P65 and interference of P65-mediated transcription in the nucleus [39]. Based on the observation of the blocking effect from SB203580, we speculated that P38 might act as an upstream signal to regulate the phosphorylation of P65 and modulate its downstream gene (TNF-α) when stimulated by SFP-F2. Interruption of both signaling pathways increased the suppression of genes downstream of P65 (TNF-α, Figure 8A). Our study therefore provided a deeper insight into the mechanisms regarding the role of key proteins in the CD14/IKK and P38 MAPK axes in RAW264.7 cells under stimulation from *S. fusiforme* polysaccharides.

In summary, we have purified a fraction of *S. fusiforme* polysaccharides, which contained a relatively high amount of sulfate and exhibited good immuno-stimulating activity in RAW264.7 cells. It enhanced the immune responses in RAW264.7 cells mainly by inducing the CD14/IKK/NF-κB and P38/NF-κB signaling pathways.

## 4. Materials and Methods

### 4.1. Chemicals and Reagents

*Sargassum fusiforme* was collected from Dongtou, Zhejiang province (Wenzhou, China). RAW264.7 macrophages were purchased from the cell bank of the Chinese Academic of Sciences (Shanghai, China). Dulbecco’s modified Eagle Medium (DMEM) and fetal bovine serum (FBS) were obtained from Gibco-BRL (Gaithersburg, MD, USA). IKK-16 (IKK Inhibitor VII), BAY 11-7082 (IκBα phosphorylation [40], E2-conjugating enzymes [41] inhibitor), and SB203580 (P38 MAPK inhibitor) were obtained from Selleck. These drugs were each dissolved in dimethyl sulphoxide (DMSO) to yield stock solutions of 1 mM. The stock solutions were diluted appropriately in DMEM medium to working concentrations. The final concentration of DMSO in the cultures was 0.02% (*v*/*v*). Unless otherwise stated, LPS and SFP-F2 were routinely dissolved in DMEM medium. ELISA kits for cytokine detection were purchased from eBioscience (San Diego, CA, USA). PDTC (IκBα-phosphorylation-Inhibitor) was purchased from Sigma (Shanghai, China). Anit-CD14 antibody was purchased from Biolegend (San Diego, CA, USA). PDTC and anti-CD14 antibody were dissolved in DMEM medium Antibodies against IκBα, Phospho-IκBα, NF-κB P65, and Phospho-NF-κB P65 were purchased from Cell Signaling Technology (Beverly, MA, USA). Antibody against β-actin was purchased from R&D Systems, and antibody against P38 and goat anti-mouse and rabbit IgGs were purchased from Beyotime (Shanghai, China). Antibodies against JNK, ERK1/2, p-P38, p-JNK, and p-ERK1/2 were purchased from Abcam (Cambridge, UK). All other chemical reagents used in this experiment were of analytical grade.

### 4.2. Extraction and Purification of Polysaccharide Fraction

*Sargassum fusiforme* was washed with distilled water and dried at 45 °C. Polysaccharide was extracted from dried *S. fusiforme* as previously described [42] to yield a crude extract. This crude polysaccharide extract was subsequently decolorized through DEAE-cellulose anion exchange chromatography (Shanghai, China). The decolorized extract was subjected to ultrafiltration and then concentrated by lyophilization to yield the decolorized crude polysaccharide, referred to as SFP [6]. SFP was subsequently subjected toanion-exchange chromatography performed with a DEAE-Sepharose CL-6B column (21.6 mm × 126 mm) followed by gel-filtration chromatography carried out with a Sephacryl^TM^ S-400 HR chromatography column (19 mm × 900 mm) (GE Healthcare, Shanghai, China). The purified fraction of the polysaccharide, designated as SFP-F2, was concentrated by ultra-filtration and lyophilized.

### 4.3. Physicochemical Characterization Analysis

The total carbohydrate content of SFP-F2 was determined by the phenol-sulfuric acid method. Uronic acid content and protein content were determined by the *M-*hydroxydiphenyl method and Bradford assay, respectively. The average molecular weight and molar ratio of the monosaccharide composition of SFP-F2 were determined as previously described [43]. An ISC-100 ion chromatography system coupled to a Shodex IC SI-52 4E column (4.0 × 250 mm) was used to determine the sulfate content [44].

The polysaccharide samples were mixed with KBr powder and pressed into pellets for FT-IR analysis, which was carried out using an Infrared Spectrometer TRENSOR 27 (Bruker Daltonics, Ettlingen, German) at the frequency range of 4000 to 400 cm^−1^.

### 4.4. RAW 264.7 Cell Viability Assay

RAW264.7 cells were seeded in a 96-well plate and cultured in DMEM medium. The cells were then treated with LPS or different concentrations of SFP-F2 for 12 h. After that, the viability of the cells was determined by MTT assay as described previously [7] and expressed as percentage relative to the control (non-treated cells), which was taken as 100%.

### 4.5. Cytokine Assay

RAW264.7 cells were treated in the same way as 4.4, and the supernatant was collected and the concentrations of TNF-α, IL-1β, and IL-6 were measured with ELISA kits (eBioscience, San Diego, CA, USA) according to the manufacturer’s protocol.

### 4.6. Reverse Transcription-Polymerase Chain Reaction (RT-PCR)

Total RNA was isolated from the cells with trizol reagent (Invitrogen Ltd., Paisley, UK) according to the manufacturer’s instructions. The cDNA was synthesized using a GoScript strand cDNA synthesis kit (Rainbio, Shanghai, China). The reaction mixture contained 1 μL of cDNA (800 ng), 5 μL of 2 × Tap PCR master Mix (Hbibio, Beijing, China), 0.5 μL of forward primer, 0.5 μL of reverse primer and 3 μL of DEPC-H_2_O. Amplification was carried out with a Veriti PCR instrument under the following conditions: an initial step at 94 °C for 1 min, followed by 27 cycles of 94 °C for 30 s, annealing at 60 °C for 30 s, and extension at 72 °C for 90 s, with a final extension step at 72 °C for 15 min. The amplified products were examined by electrophoresis in 0.8% agarose gel and the intensity of each band was quantified by ImageJ software, stained by GoodView^TM^. The amplification of β-actin was used as an internal standard. The sequences of the primers used in the present study are as follows:

TNF-α, F: ACTTCGGGGTGATCGGTC, R: TGTCTTTGAGATCCATGCCG;
IL-1β, F: GAGCTTCAGGCAGGCAGTAT, R: TGGGTGTGCCGTCTTTCATT;
IL-6, F: CACTTCACAAGTCGGAGGC, R: GCACTAGGTTTGCCGAGTAGA;
β-actin, F: CCCTGTATGCCTCTGGTCGT, R: CACCAGACAGCACTGTCTTGG.

### 4.7. Western Blot

RAW264.7 cells were treated with 1 μg/mL LPS, different concentrations of SFP-F2 or a series of inhibitors. The cells were collected, washed twice with ice-cold PBS, and then lysed in RIPA lysis buffer [9]. Equal amounts of total protein from the different cell extracts were separated by SDS-PAGE using 8–10% gels. The proteins in the gels were subsequently transferred onto a PVDF membrane. The membrane was blocked with 5% non-fat milk and probed with the appropriate primary antibody. The blot was then incubated with HRP-conjugated secondary antibody and positive signals were detected by the enhanced chemiluminescence (ECL) reagent (SaiZhi, Beijing, China). Data were quantified using the Amersham Imager 600 (GE, MA, USA).

### 4.8. Statistical Analysis

Data were expressed as means ± standard deviations (SD). Comparison of the data from multiple groups was performed using one-way analysis of variance (ANOVA) followed by Tukey test or the Tukey-Kramer test. GraphPad Prism version 5.00 (GraphPad, San Diego, CA, USA) and SPSS were used to plot and analyze the data. *p* < 0.05 was considered statistically significant. The significant differences were indicated by different letters (*p* < 0.05); the same letters meant no statistical significances.

## Figures and Tables

**Table 1:** Chemical composition and structural characteristics of SFP-F2.

Fraction	Yield (%)	Mw (kDa)	Sugar (%)	UA (%)	Protein (%)	SO_4_^2−^ (%)	Monosaccharide Composition (%)				
							Man	GalA	Gal	Xyl	Fuc
SFP-F2	1.75	24	62.9	14.7	0.4	27.7	2.4	0.7	13.3	3.0	80.6

Yield = Mass of fraction/*S. fusiforme* powder; Mw = Molecular weight; UA = Uronic acid.
